# Supplementary material for: The evolutionary dynamics between viral mimics and host proteins
Source: Mol Syst Biol. 2026 Mar 20;22(6):902–27. doi: 10.1038/s44320-026-00200-1 (PMC13230584; doi:10.1038/s44320-026-00200-1)
Supplement: Supplementary file 4 — Appendix [file 44320_2026_200_MOESM4_ESM.pdf]

Appendix for “The evolutionary dynamics between viral mimics and host proteins”

Table of contents

Appendix Figure S1: Violin plots showing the numbers of tissues expressing genes, across healthy adult human tissues for each gene group – page 2

Appendix Figure S2: Box plots showing level of fold complexity and percentage of disordered residues of viral mimicking and non-mimicking proteins – page 3

Appendix Figure S3: Bar plots showing percentage of genes with signatures of positive selection (PSGs) in different viral gene groups. – page 4

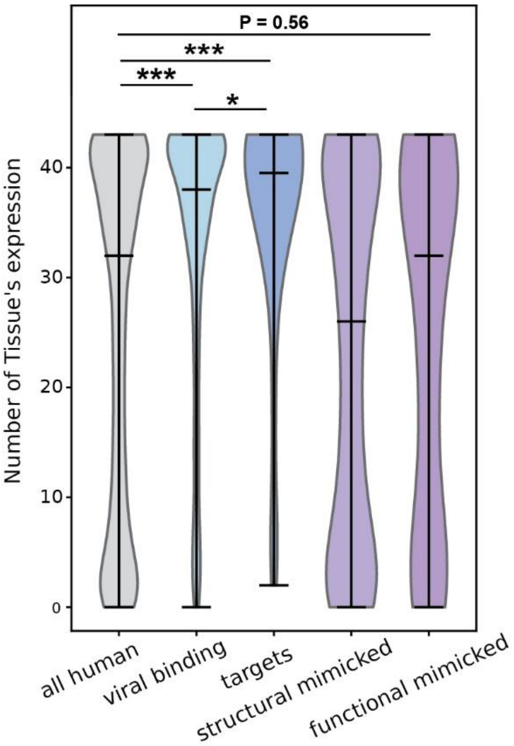

Appendix Figure S1: Violin plots showing the numbers of tissues expressing genes, across healthy adult human tissues for each gene group: all human proteins (9,173 proteins), human proteins experimentally known to interact with at least one viral protein from the set of the five dsDNA viruses used in this study (viral binding, 3,489 proteins), human proteins known to interact with viral mimics and host mimicked proteins(mutual targets, 58 proteins), human proteins that have at least one structural homolog in the five viral proteomes used in this study (structural mimicked, 586 proteins), a subset of structural mimicked that are also known to interact with the same target as viral mimicking proteins (functional mimicked, 106 proteins). Gene is considered to be expressed in a given tissue if it has a TPM (transcript per million) value of at least. Groups were compared using Mann-Whitney test and corrected by FDR. $***$ $P < 0.001$, $**$ $P < 0.01$, $*$ $P < 0.05$.

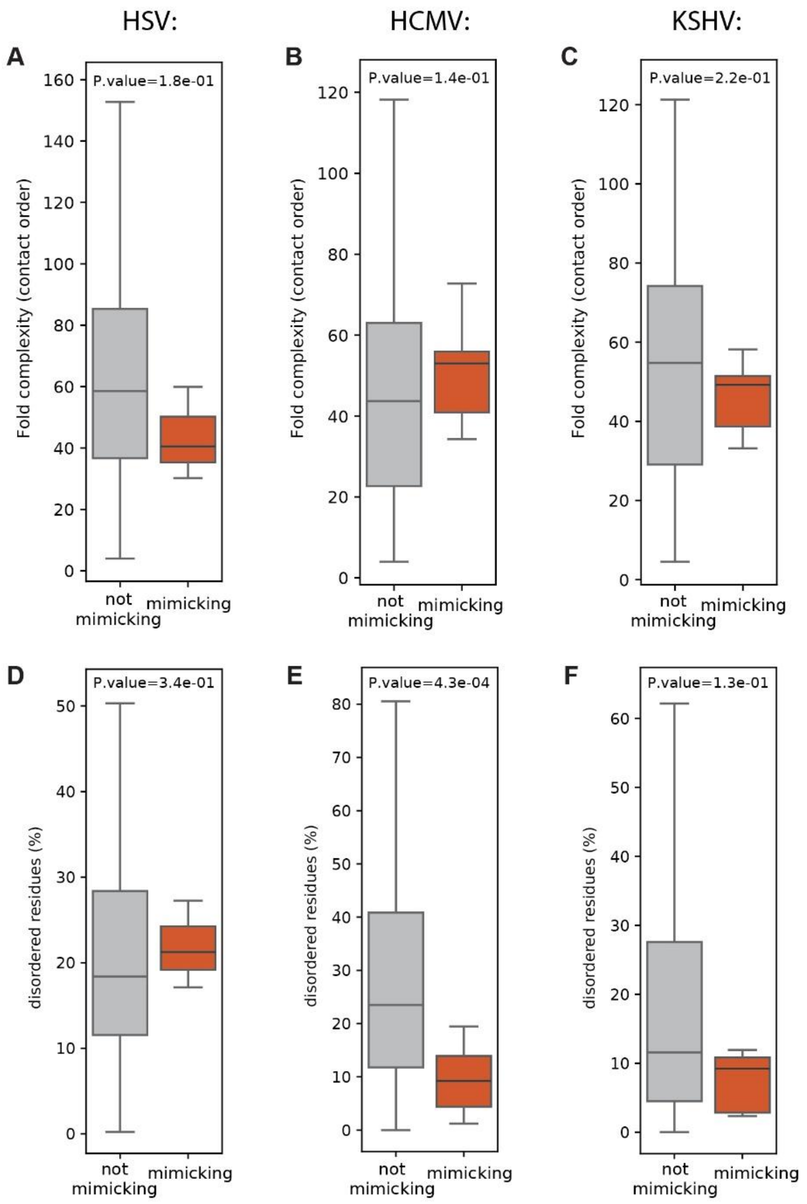

Appendix Figure S2: Box plots showing level of fold complexity and percentage of disordered residues of viral mimicking and non-mimicking proteins: (A-C) Fold complexity values of viral mimicking versus not mimicking proteins as measured using contact order, values are taken from Fuchs et al., 2025 (left to right: HSV1, HCMV, KSHV). (D-F) Disorder residues percent of viral mimicking versus not mimicking proteins, as measured using AlphaFold residue pLDDT scores, values are taken from Fuchs et al., 2025 (left to right: HSV1, HCMV, KSHV).

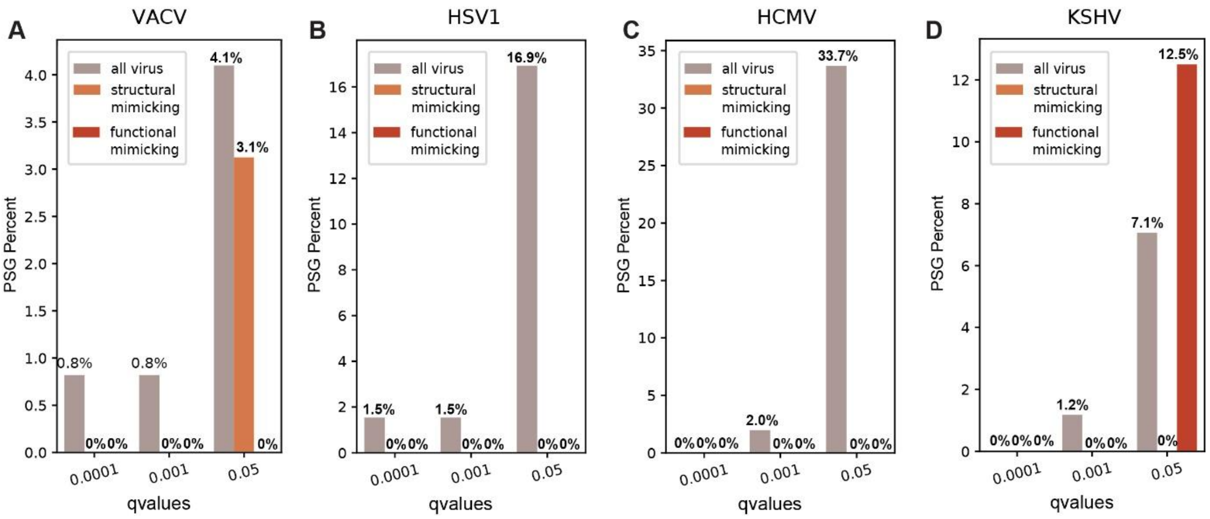

Appendix Figure S3: Bar plots showing percentage of genes with signatures of positive selection (PSGs) in different viral gene groups: Protein sets are from: (A) VACV (representing orthopoxviruses), (B) HSV-1 rSimplexviruses), (C) HCMV (cytomegaloviruses), and (D) KSHV (rhadinoviruses). Comparing sets of all viral proteins, structural-mimicking and functional-mimicking protein (see protein definitions and numbers in Fig S5). PSGs were identified using site-models (in A) and branch-site models (in B-D), and based on a statistical significance threshold. Percentage of PSGs are shown with the following thresholds: FDR-corrected P-values of 0.0001, 0.001 and 0.05. Statistical enrichment (or depletion) was computed for each gene subset with respect to the group of all viral genes using Fisher's exact test and corrected by FDR. None of the comparisons resulted in a significant depletion (i.e., all P-values > 0.05). With the exception of KSHV analysis using a threshold of PSG detection of FDR-corrected P-value=0.05 (right- most section in panel D), in all analyses and thresholds the functional- and structural-mimicking proteins have lower fraction of PSGs than the set of all viral proteins.
