# Supplementary material for: The evolutionary dynamics between viral mimics and host proteins
Source: Mol Syst Biol. 2026 Mar 20;22(6):902–27. doi: 10.1038/s44320-026-00200-1 (PMC13230584; doi:10.1038/s44320-026-00200-1)
Supplement: Supplementary file 15 — Expanded View Figures [file 44320_2026_200_MOESM15_ESM.pdf]

Expanded View Figures

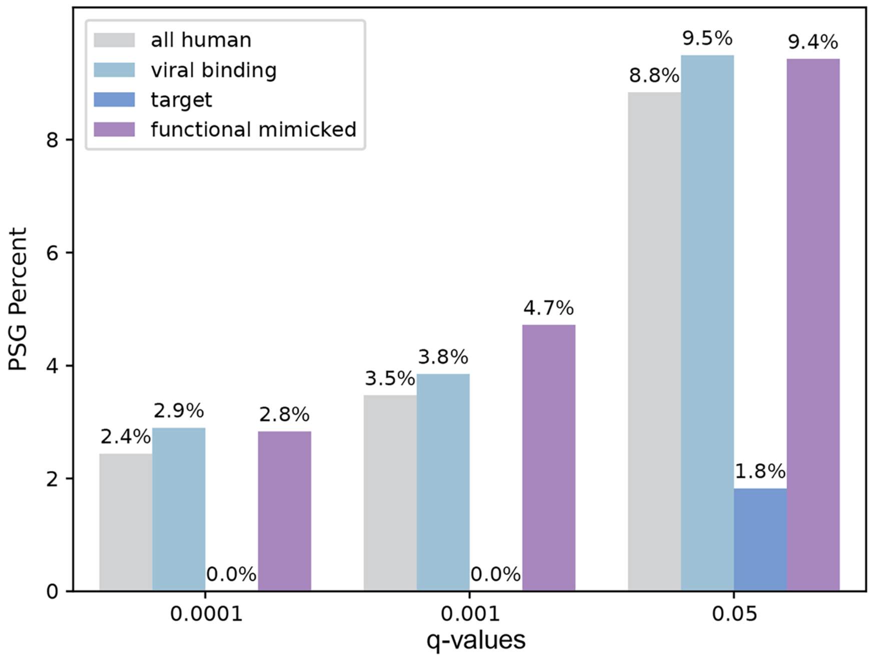

Figure EV1. Bar plots showing percentage of genes within each group, with signatures of positive selection (PSGs), as follows.

All human proteins (9474 proteins), human proteins experimentally known to interact with at least one viral protein from the set of the five dsDNA viruses used in this study (viral binding, 3599 proteins), human proteins known to interact with viral mimics and host-mimicked proteins (mutual targets, 59 proteins), human proteins that have at least one structural homolog in the five viral proteomes used in this study (structural mimicked, 605 proteins), a subset of structural mimicked that are also known to interact with the same target as viral mimicking proteins (functional mimicked, 111 proteins). The evolutionary rate is relative across all proteins residues (5,751,638 residues in total) and is computed based on substitutions across a set of 10 one-to-one orthologs in primates, using the ratio between the number of non-synonymous and synonymous substitutions (dN/dS values, left). PSGs were identified based on a likelihood ratio test between two models (M8 versus M8A), and based on a statistical significance threshold. Three different thresholds are shown (from left to right, FDR-corrected *P* values of 0.0001, 0.001 and 0.05). Percentage of PSGs with FDR-corrected *P* value of 0.01 is shown in Fig. 2B. Statistical enrichment (or depletion) was computed for each gene subset with respect to the group of all human genes using Fisher's exact test and corrected by FDR. None of the comparisons resulted in a significant *P* value (i.e., all *P* values > 0.05). The total numbers of PSGs in each of the categories - all human, viral binding, target and functional mimicked, are in: FDR-corrected *P* values of 0.0001: 211,103,0,3; and FDR-corrected *P* values of 0.001: 301,137,0,5; and FDR-corrected *P* values of 0.05: 766,338,1,10, respectively. /ppImages were created in BioRender. (2025) https://BioRender.com/1m65sw7.

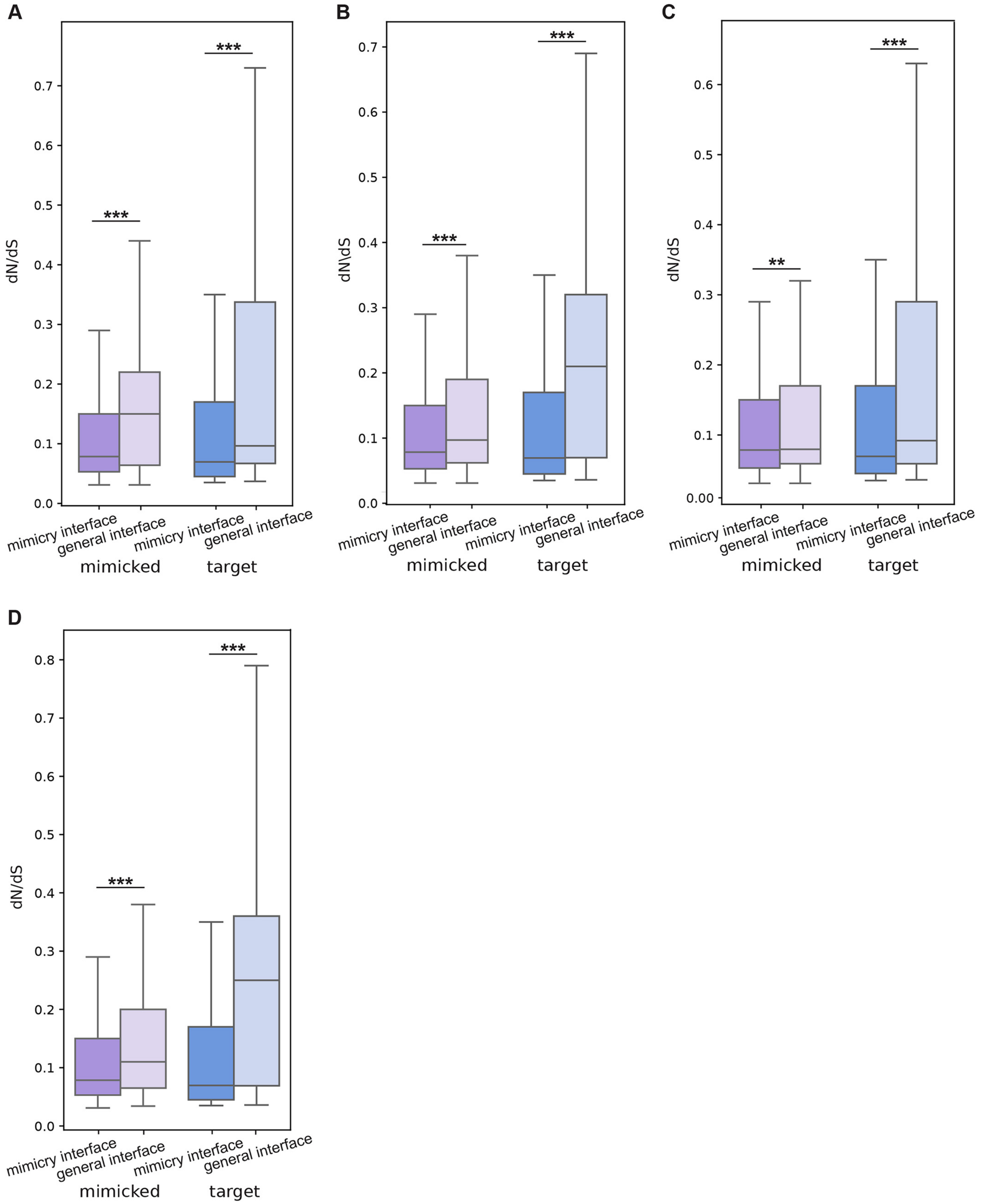
A
B
C
D
dN/dS
***
**
0.0
0.1
0.2
0.3
0.4
0.5
0.6
0.7
0.8
0.00
mimicry interface
general interface
mimicked
target

 © The Author(s)

Figure EV2. Comparison of evolutionary rates between mimicry interface and general interface residues, in mimicked and target proteins.

As in Fig. 3C, with the mimicry-interface residues predicted based on the complexes between host-mimicked and target proteins using the CSU method, and the general-interface residues predicted by ScanNet, but with different set of parameters: (**A**) ScanNet score of 0.7 and above is used to determine interface residues, only residues predicted to be ordered regions are considered (unlike in Fig. 3C, where both disordered and ordered residues were considered). *P* values for comparisons of mimicked general interface vs. mimicked mimicry interface, and target general interface vs. target mimicry interface are 1.44e-06 and 2.33e-10, respectively. *N* of residues in mimicked–general interface, mimicry interface and target–general interface, mimicry interface are 145,642,218,762 residues, respectively. (**B**) ScanNet score of 0.5 and above is used to determine interface residues, all residues (both ordered and disordered) are considered. *P* values for comparisons of mimicked general interface vs. mimicked mimicry interface, and target general interface vs. target mimicry interface are 2.63e-07 and 1.70e-42, respectively. *N* of residues in mimicked–general interface, mimicry interface and target–general interface, mimicry interface are 1014,642,1147,762 residues, respectively. (**C**) ScanNet score of 0.5 and above is used to determine interface residues, only residues predicted to be ordered regions are considered. Groups were compared using Mann–Whitney test and corrected by FDR. *P* values for comparisons of mimicked general interface vs. mimicked mimicry interface, and target general interface vs. target mimicry interface are 1.29e-03 and 5.70e-09, respectively. *N* of residues in mimicked general interface, mimicry interface and target general interface, mimicry interface are 478,642,437,762 residues, respectively. (**D**) ISPRED4 probability of 0.7 and above is used to determine interface residues (all residues were considered). $^{***}P < 0.001$, $^{**}P < 0.01$, $^{*}P < 0.05$. Boxplots in (**A–D**) represent the median, first quartile and third quartile with lines extending to the furthest value within 1.5 of the interquartile range (IQR). *P* values for comparisons of mimicked general interface vs. mimicked mimicry interface, and target general interface vs. target mimicry interface are 2.58e-14 and 4.90e-55, respectively. *N* of residues in mimicked–general interface, mimicry interface and target–general interface, mimicry interface are 1335,642,1525,762 residues, respectively.

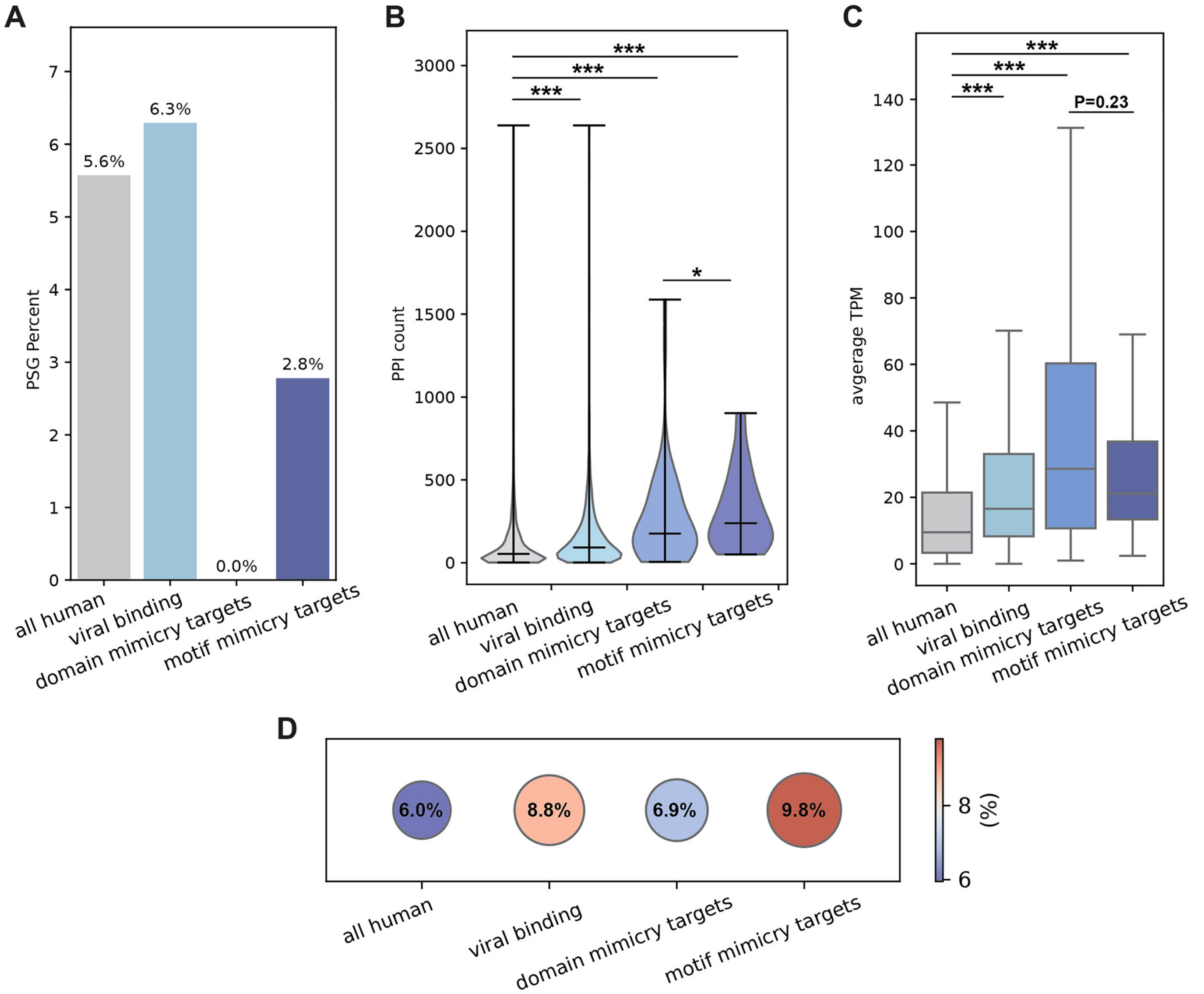

Figure EV3. Comparison of evolutionary, functional and cellular characteristics between different groups of human proteins, including targets of motif mimicry.

(**A**) Bar plots showing percentage of genes within each group, with signatures of positive selection (PSGs), as follows: all human proteins (9173 proteins), human proteins experimentally known to interact with at least one viral protein from the set of the five dsDNA viruses used in this study (viral binding, 3489 proteins), human proteins known to interact with viral domain mimics and host-mimicked domains (domain-mimicry targets, 58 proteins), human proteins known to interact with viral motif mimics and host-mimicked motifs (motif-mimicry targets, 49 proteins). Statistical enrichment (or depletion) was computed for each gene subset with respect to the group of all human genes using Fisher's exact test and corrected by FDR. None of the comparisons resulted in a significant *P* value, except for viral-binding versus all human proteome. (**B**) Violin plots showing the number of within-host PPIs for the sets of proteins defined in (**A**) (each filled area extends to represent the entire data range). *P* values for comparisons of all vs. viral binding, domain mimicry targets, motif mimicry targets and domain mimicry targets vs. motif mimicry targets are 1.67e-86, 8.733e-12, 2.775e-17, 3.99e-02, respectively. (**C**) Boxplots showing the distributions of average gene-expression levels across healthy adult human tissues for each gene group described in (**A**). *P* values for comparisons of all vs. viral binding, domain mimicry targets, motif mimicry targets, and domain mimicry targets vs. motif mimicry targets are 4.4e-125, 6.48e-009, 5.934e-008, 2.266e-001, respectively. (**D**) Dotplots showing the fraction of essential genes within each set of genes defined in A. None of the comparisons resulted in a significant, except for viral-binding versus all human proteome (*P* value = 5.4×10^{-8}). In all panels: $^{***}P < 0.001$, $^{**}P < 0.01$, $^{*}P < 0.05$. Boxplots in (**C**) represent the median, first quartile and third quartile with lines extending to the furthest value within 1.5 of the interquartile range (IQR).

 © The Author(s)

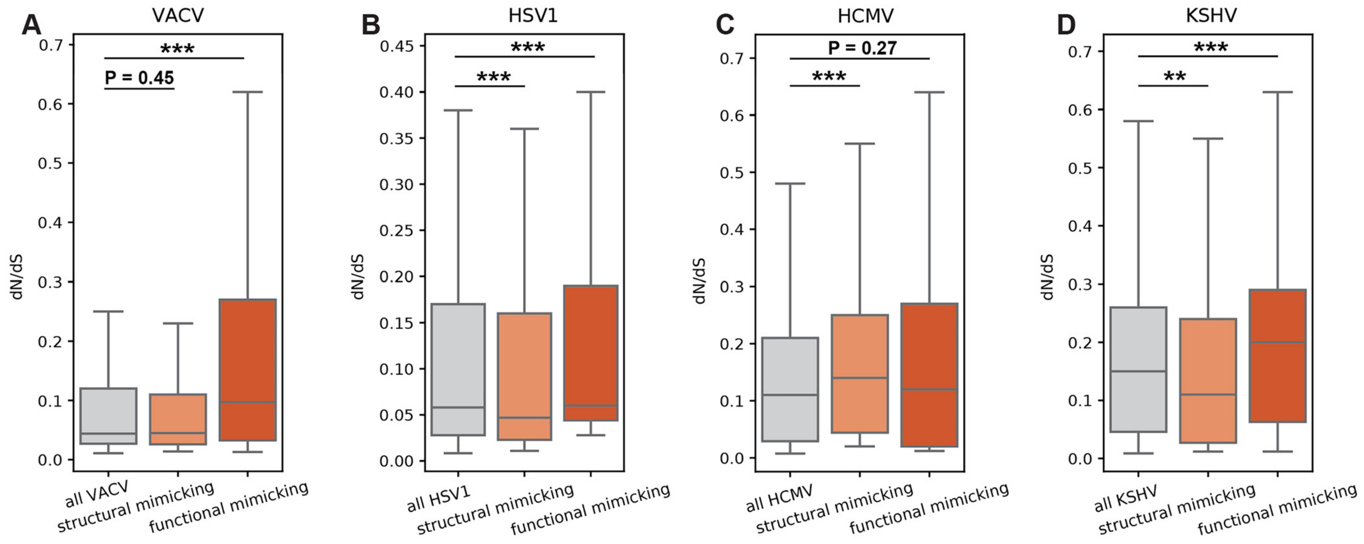

Figure EV4. Boxplots showing the distribution of evolutionary rates of different viral protein sets, comparing rates between all viral proteins, structural-mimicking, and functional-mimicking proteins.

(**A**) VACV (representing orthopoxviruses; all proteins—113, structural mimicking—33, functional mimicking—3), *P* values for comparisons of all VACV vs. structural mimicking, functional mimicking are 0.45, 8.618e-41, respectively. *N* of residues for all VACV, structural mimicking, functional mimicking are 33320, 10093, 1052, respectively. (**B**) HSV1 (simplexviruses—all proteins—69, structural mimicking—5, functional mimicking—3), *P* values for comparisons of all HSV1 vs. structural mimicking, functional mimicking are 7.109e-25, 2.89e-21, respectively. *N* of residues for all HSV, structural mimicking, functional mimicking are 36219, 3214, 1221 residues, respectively. (**C**) HCMV (cytomegaloviruses—all proteins—78, structural mimicking—4, functional mimicking— 2), *P* values for comparisons of all HCMV vs. structural mimicking, functional mimicking are 2.509e-34, 2.721e-01, respectively. N of residues for all HCMV, structural mimicking, functional mimicking are 38217, 2192, 750 residues, respectively. (**D**) KSHV (rhadinoviruses—all proteins—98, structural mimicking—5, functional mimicking—8). *P* values for comparisons of all KSHV vs. structural mimicking, functional mimicking are 2.034e-12, 9.518e-26, respectively. *N* of residues for all KSHV, structural mimicking, functional mimicking are 32256, 1849, 2253 residues, respectively. Evolutionary rates across each lineage were computed using Selecton and MSAs based on one-to-one viral orthologs, as described in Methods. In these plots, viral proteins that have significant structural domain homology to at least one human protein were considered as mimicking, and are here partitioned into structural- and functional-mimicking, where they are mutually exclusive and the partition is based on whether or not the viral-mimicking protein has a shared interactor with the host-mimicked protein. Groups were compared using Mann–Whitney test and corrected by FDR. ***$P < 0.001$, **$P < 0.01$, *$P < 0.05$. Boxplots in (**A–D**) represent the median, first quartile and third quartile with lines extending to the furthest value within 1.5 of the interquartile range (IQR).

© The Author(s)

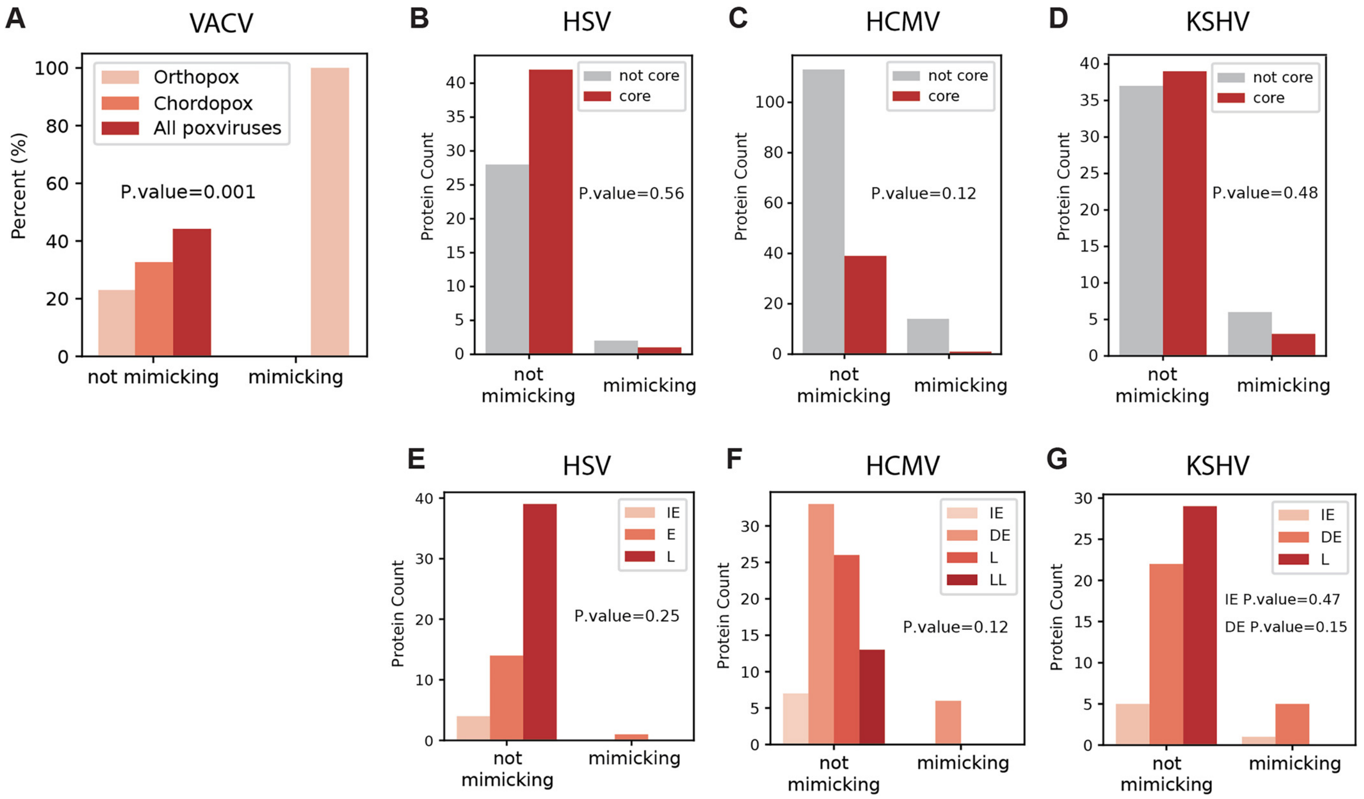

Figure EV5. Occurrence of mimicking and non-mimicking proteins in various functional groups.

(**A**) Occurrence of VACV proteins, partitioned based on functional versus non-functional mimicking proteins in different evolutionary ages. (**B–D**) Occurrence of herpesvirus proteins, partitioned based on functional versus non-functional mimicking proteins, and partitioned based on core vs non-core proteins. (**E–G**) Occurrence of herpesvirus proteins, partitioned based on functional versus non-functional mimicking proteins, and partitioned based on temporal gene expression.

 © The Author(s)
